# Role of the gut microbiota in hematologic cancer

**DOI:** 10.3389/fmicb.2023.1185787

**Published:** 2023-08-25

**Authors:** Patricia Guevara-Ramírez, Santiago Cadena-Ullauri, Elius Paz-Cruz, Rafael Tamayo-Trujillo, Viviana A. Ruiz-Pozo, Ana Karina Zambrano

**Affiliations:** Centro de Investigación Genética y Genómica, Facultad de Ciencias de la Salud Eugenio Espejo, Universidad UTE, Quito, Ecuador

**Keywords:** hematologic cancer, leukemia, lymphoma, microbiota, multiple myeloma

## Abstract

Hematologic neoplasms represent 6.5% of all cancers worldwide. They are characterized by the uncontrolled growth of hematopoietic and lymphoid cells and a decreased immune system efficacy. Pathological conditions in hematologic cancer could disrupt the balance of the gut microbiota, potentially promoting the proliferation of opportunistic pathogens. In this review, we highlight studies that analyzed and described the role of gut microbiota in different types of hematologic diseases. For instance, myeloma is often associated with *Pseudomonas aeruginosa* and *Clostridium leptum*, while in leukemias, *Streptococcus* is the most common genus, and *Lachnospiraceae* and *Ruminococcaceae* are less prevalent. Lymphoma exhibits a moderate reduction in microbiota diversity. Moreover, certain factors such as delivery mode, diet, and other environmental factors can alter the diversity of the microbiota, leading to dysbiosis. This dysbiosis may inhibit the immune response and increase susceptibility to cancer. A comprehensive analysis of microbiota-cancer interactions may be useful for disease management and provide valuable information on host-microbiota dynamics, as well as the possible use of microbiota as a distinguishable marker for cancer progression.

## Introduction

Hematologic malignancies are characterized by the uncontrolled growth of hematopoietic and lymphoid cells, resulting in decreased immune system efficacy (Méndez-Ferrer et al., 2020). Hematologic neoplasms account for 6.5% of all cancers worldwide (De Moraes Hungria et al., 2019; Kocarnik et al., 2022). The World Health Organization (WHO) classifies hematologic malignancies based on morphology, immunophenotype, genetics, and clinical features (Khoury et al., 2022). The most common subtypes include leukemia, Hodgkin’s lymphoma (HL), non-Hodgkin’s lymphoma (NHL), and multiple myeloma (MM) (Keykhaei et al., 2021). Hematologic diseases have been associated with genetic factors and alterations of the immune system. However, several studies also suggest a potential correlation between hematologic cancers and alteration in the microbiota. For instance, research shown that the growth of gastric mucosa-associated lymphoid tissue (MALT) lymphoma tumors can be stimulated by signaling antigens released by the bacterium *Helicobacter pylori* (*H. pylori*), highlighting a possible link between bacteria and MALT lymphoma (Ferreri et al., 2013; Kuo and Cheng, 2013; Portlock et al., 2015).

The human gut microbiota (GM) is a population of microorganisms, including bacteria, archaea, fungi, protozoa, and viruses, that coexist within the intestinal tract (D’Angelo et al., 2021). Furthermore, these microorganisms produce metabolites such as short-chain fatty acids (SCFAs), which could have anti-carcinogenic properties. The most predominant SCFAs, acetate, propionate, and butyrate, play crucial roles in ion absorption and intestinal motility (Jasiński et al., 2021). In particular, butyrate has been studied for its anti-inflammatory properties (Ubeda et al., 2010; Canani et al., 2011; Zimmerman et al., 2012; Bin et al., 2021). However, conflicting findings suggest that the effects of butyrate on cell proliferation vary, depending on factors such as time, cell type, and concentration; it could either promote or prevent cell proliferation. Nonetheless, it has been proposed that excessive butyrate production following dysbiosis and inflammation may promote tumor proliferation, potentially outweighing its beneficial properties (Donohoe et al., 2012).

Metagenomics and metabolomics analyses have provided valuable insights into the role of intestinal microbiota in malignant neoplasms (Frankel et al., 2017). These studies suggest that pathological conditions in hematologic cancer (HC) can lead to dysbiosis, which is an imbalance of the microbiota (Ahmed et al., 2020; Dutta and Lim, 2020; Tsvetikova and Koshel, 2020; Zheng et al., 2020; Abdelazeem et al., 2021). Imbalances in the microbiota can inhibit the colonization of beneficial probiotic bacteria, promote harmful enteropathogens proliferation, and alter cytokine signaling, thus affecting the immune system (Alexander et al., 2017). In this review, we highlight studies that analyzed the role of GM in different types of hematologic diseases, especially leukemias, lymphomas, and myelomas. Additionally, we describe the factors that can alter the human gut microbiota and its correlation with hematologic cancer predisposition and progression.

## Gut microbiota and hematologic diseases

Hematologic diseases have been associated with dysbiosis, leading to a limited capacity of the microbiota’s metabolites to modulate inflammatory processes, and disrupting intestinal homeostasis. Understanding the relationship between the host and gut microbiota is crucial. Germ-free mice experiments have shown that certain bacteria, such as *Bacteroides* and *Escherichia* spp., could have an immunogenic effect by stimulating the production of immunoglobulin A (IgA) plasmacytes (Moreau et al., 1978; Strauch et al., 2005). The microbiota interacts with the immune system via the intestinal epithelium, which comprises enterocytes, goblet cells, neuroendocrine cells, tuft cells, Paneth cells, and Microfold cells (M cells), plays an essential role in innate immunity and host defense (Allaire et al., 2018).

Peyer’s patches are clusters of lymphoid tissue that line the walls of the small intestine. They contain immune cells such as innate lymphoid cells (ILCs), natural killer (NK) cells, T and B lymphocytes, and M cells (Elemam et al., 2017). Pattern recognition receptors (PRRs), including Toll-like receptors (TLRs) and Nod-like receptors (NLRs), are expressed by both epithelial and immune cells. These receptors can recognize pathogen-associated molecular patterns (PAMPs) and damage-associated molecular patterns (DAMPs) (Rankin et al., 2013). Remarkably, a study in mice suggests that gut microbiota manipulation can modulate cancer immunotherapy by increasing T cells within the tumor microenvironment (Sivan et al., 2015). GM has been linked to immunological response because microorganisms can facilitate the transport of macromolecules and antigens through the gut epithelium.

Moreover, flagellin is the primary component of the bacterial flagellum; it mediates the interaction between the intestinal epithelium and host immunity. Flagellin can be recognized by TLR5, found in B-cells and CD4+ T-cells. Differentiated B-cells produce IgA that neutralizes the pathogen and prevents subsequent infection (Eaves-Pyles et al., 2011; Haiko and Westerlund-Wikström, 2013). Generally, TLRs activation by antigens from the normal gut microbiota signals the inhibition of inflammatory reactions, which is necessary to maintain intestinal homeostasis. NLRs recognize specific microbial molecules and initiate the formation of inflammasomes, which act as sensors for damage-associated patterns (Lavelle et al., 2010; Parlato and Yeretssian, 2014). Thus, immune dysregulation in hematologic diseases could alter the interaction with the microbiota, inhibiting the role of its metabolites and leading to an increased vulnerability to infections and a rise in the severity of hematological cancer.

## Factors associated with gut microbiota composition and hematologic cancer

**Figure 1:**
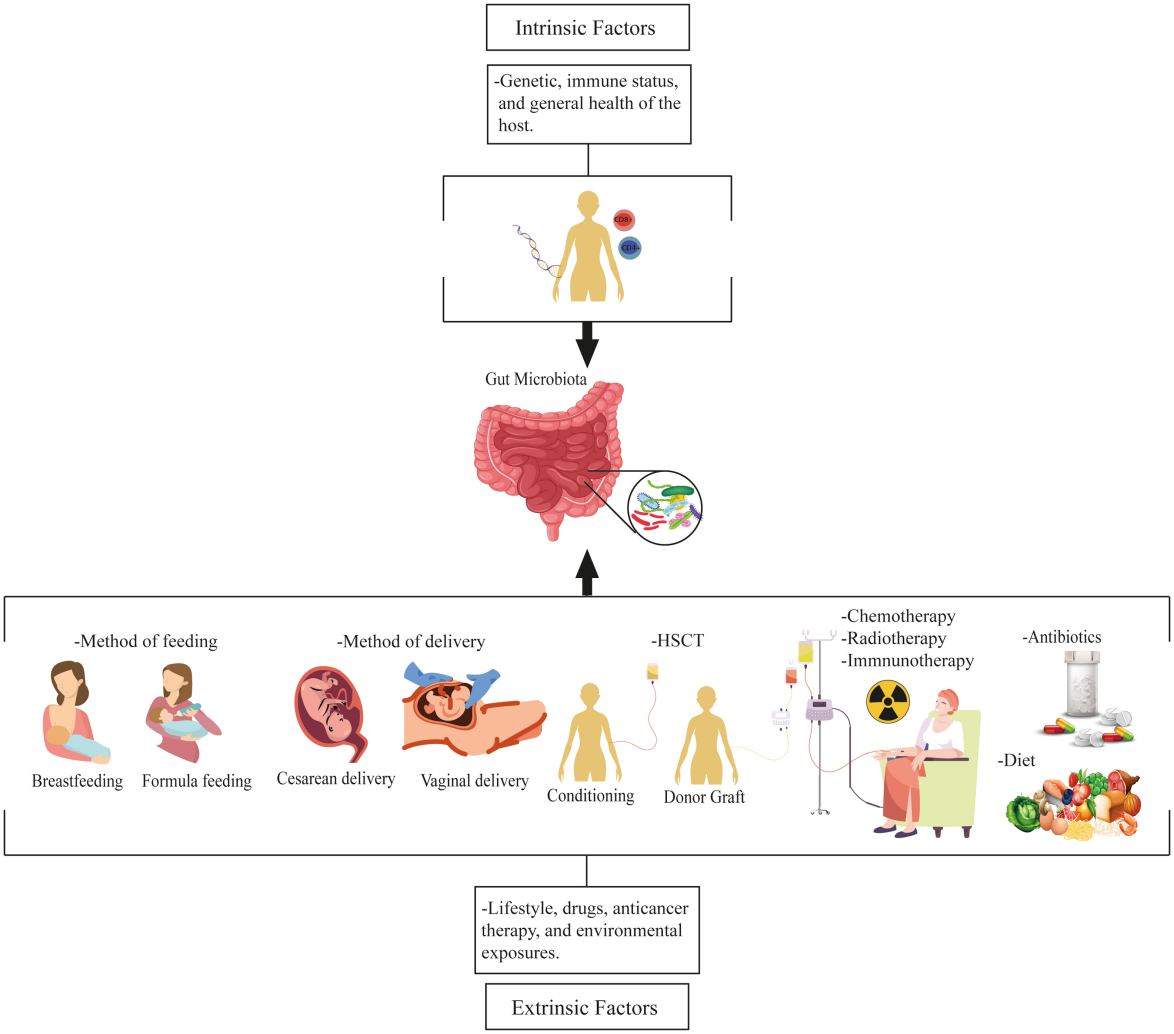
Factors influencing the gut microbiota and its relationship to hematologic malignancies. These factors are intrinsic and extrinsic. Intrinsic factors include genetics, immune status, and overall health, whereas extrinsic factors include nutrition, lifestyle, medications, anticancer therapy, and environmental exposures that affect the gut microbiota. HSCT, Hematopoietic stem cell transplantation.

The interactions between the microbiome and hematologic cancer are influenced by intrinsic and extrinsic factors. Intrinsic factors, such as genetics, immune status, and overall health, can shape both the composition and functionality of the gut microbiota. Genetic variations in host genes can influence the expression of microbial receptors, impacting the colonization and survival of specific microbial species. Immune dysregulation can lead to microbial imbalances contributing to carcinogenesis (Rahman et al., 2022). Extrinsic factors, such as nutrition, lifestyle, drugs, anticancer therapy, and environmental exposures, also influence the gut microbiota. Physical exercise, stress, diet, type of delivery, pollution, and chemicals indirectly impact the gut microbiota through their effects on human physiology and metabolism (Bajinka et al., 2020). Altogether, these variables alter the gut microbial ecosystem, increasing the host’s susceptibility to hematopoietic malignancies (Figure 1; Uribe-Herranz et al., 2021).

### Method of delivery

The type of delivery can influence the diversity of the neonate’s gut microbiota. During vaginal delivery, the neonate is exposed to vaginal, perineal, and fecal flora, with the most abundant bacteria being *Lactobacillus*, *Prevotella*, *Sneathia* (Stiemsma and Michels, 2018), and *Gardnerella vaginalis* (Chen et al., 2021). Conversely, neonates born by cesarean delivery have distinct intestinal microbiota colonized by skin bacteria, such as *Staphylococcus*, *Corynebacterium*, and *Propionibacterium* (Greenbaum et al., 2018; Sędzikowska and Szablewski, 2021). Research has correlated the type of delivery with a predisposition to the development of hematologic diseases such as leukemia and HL, concluding that cesarean deliveries had higher rates of HC development compared to vaginal delivery (Momen et al., 2014; Greenbaum et al., 2018; Marcoux et al., 2022).

### Method of feeding

Breastfeeding colonizes the infant’s gut microbiome through contact with the nipple-areola and breast milk microbes. The microbiota of breastfed infants is dominated by *Bifidobacterium*, *Ruminococcus,* and *Lactobacillus* spp. In contrast, bottle-fed infants exhibit a higher prevalence of Proteobacteria, *Streptococcus*, *Bacteroides*, *Clostridium*, *Bifidobacterium,* and *Atopobium* in their microbiota. According to numerous studies, breastfeeding is important in lowering the risk of infant leukemia (Ajrouche et al., 2015; Amitay et al., 2016), while formula feeding has been associated with an increased risk of various diseases (Stiemsma and Michels, 2018; Sędzikowska and Szablewski, 2021; Su et al., 2021).

### Dietary factors

Recent studies have suggested that dietary factors can shape gut microbiota (Alexander et al., 2017; Uribe-Herranz et al., 2021). There are different diet types, depending on the country and the area (rural or urban). Certain diets are characterized by high fat and carbohydrate intake but low fiber, while others are rich in both protein and fiber. The metabolism of these foods can result in the enrichment or elimination of different bacterial populations and lead to the formation of specific metabolites (Koh et al., 2016; Li et al., 2021). Investigations found that fiber (Liu et al., 2015), oligosaccharides (Hosomi et al., 2009), glutamine (Han et al., 2016), and lactoferrin are potentially beneficial molecules during leukemia treatment because they increase the proportions of beneficial commensals (Iyama et al., 2014; Masetti et al., 2021).

### Other factors

The composition of the microbiota is influenced by various factors, including cancer treatments and therapies. One critical factor are medications, such as antibiotics, which can disrupt the balance of the gut microbiota, leading to dysbiosis that may affect cancer treatment outcomes. For example, although antibiotics are commonly administered in hematologic cancer treatment to prevent infections, they can affect bacteria such as *Faecalibacterium, Anaerostipes,* and *Blautia*, potentially disrupting the overall gut microbial ecosystem (Dunn et al., 2022; Sochacka-ćwikła et al., 2022).

Furthermore, various anticancer treatments, such as chemotherapy, radiotherapy, and immunotherapy, have a profound impact on the gut microbiota of hematologic cancer patients. Specific chemotherapeutic drugs (cladribine, vidarabine, cisplatin, and gemcitabine) may become less effective against certain bacteria, and could decrease the abundance of beneficial bacteria like *Bifidobacterium*, *Lactobacillus*, and *Faecalibacterium prausnitzii* (*F. prausnitzii*) while promoting potentially harmful bacteria, such as *Escherichia* and *Enterococcus faecium* (Zwielehner et al., 2011; Pflug et al., 2016; Dunn et al., 2022).

Additionally, hematopoietic stem cell transplantation (HSCT) can lead to changes in the microbiota and give rise to complications such as graft-versus-host disease (GVHD). Severe GVHD has been associated with an increased abundance of *Enterobacteriaceae*, while *Clostridia* have been linked to anti-inflammatory responses (Hong et al., 2021). Studies have demonstrated shifts in the microbiota during the conditioning stage, with chemotherapeutic agents damaging intestinal epithelial cells and increasing the susceptibility to bacteremia (Shono and van den Brink, 2018; Hong et al., 2021; Ingham et al., 2021; Margolis et al., 2023). The conditioning regimen used before HSCT significantly alters the gut microbiome, surpassing even the effects of the transplant itself (Jørgensen et al., 2022).

In summary, the relationship between microbiota and hematologic cancer is complex and influenced by various factors. Understanding these factors and their impact on the gut microbiota is crucial for developing personalized therapeutic strategies.

## Alteration of gut microbiota in hematologic cancer

**Figure 2:**
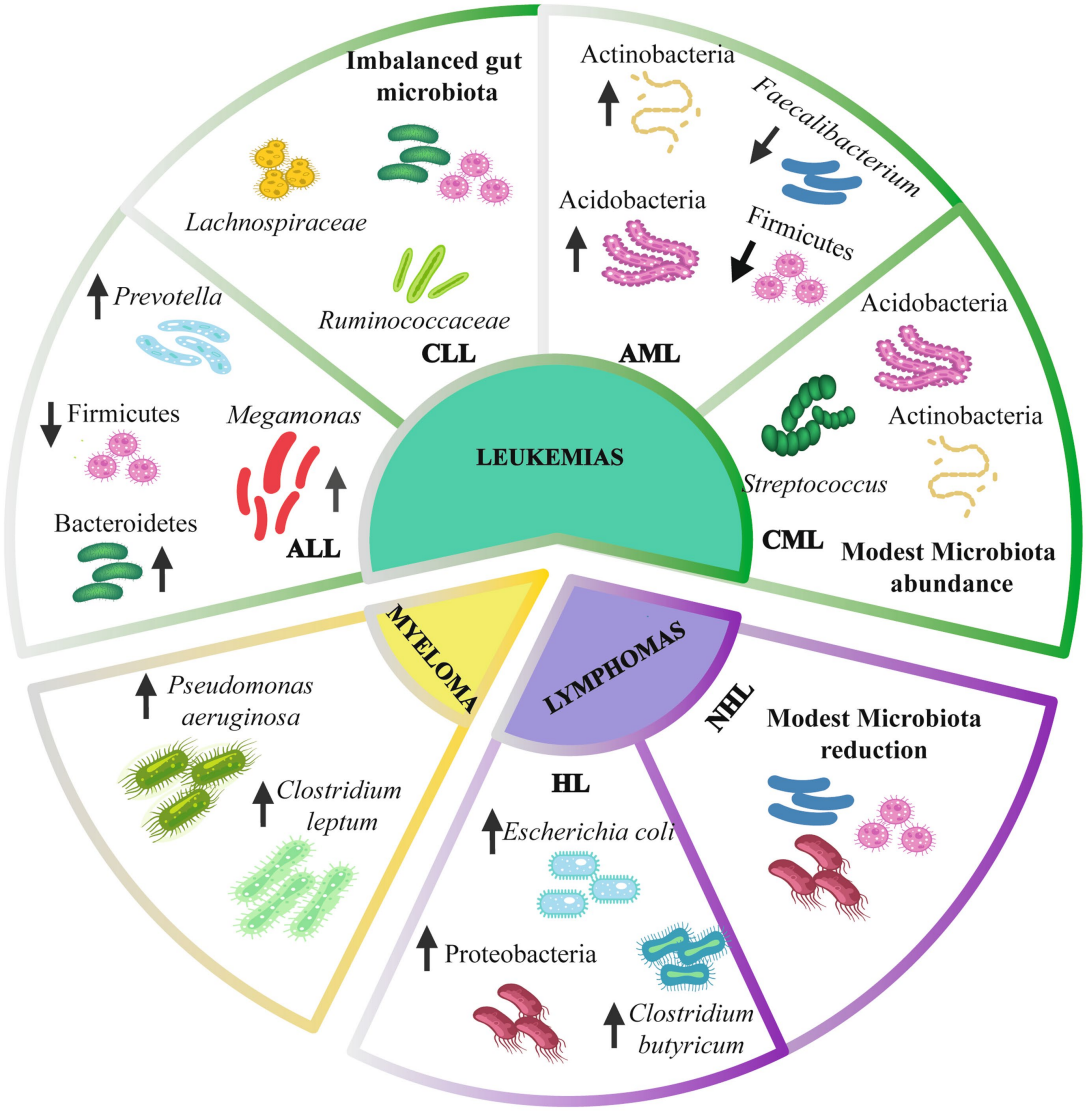
Gut microbiota composition in hematologic cancer. Leukemias have alterations of the intestinal microbiome at the phylum level, including Firmicutes, Bacteroidetes, and Actinobacteria. At the genus level, there is an alteration in *Prevotella*, *Megamonas*, *Faecalibacterium*, and *Streptococcus*. Lymphomas present a modest reduction of the intestinal microbiota, mainly an increase in *Escherichia coli* and *Clostridium butyricum*. Myeloma presents an alteration of *Pseudomonas aeruginosa* and *Clostridium leptum* species. Up arrows indicate an increase. Down arrows indicate a decrease. ALL, Acute lymphoblastic leukemia; CLL, Chronic lymphoblastic leukemia; AML, Acute myelogenous leukemia; CML, Chronic myelogenous leukemia; HL, Hodgkin lymphoma; NHL, non-Hodgkin lymphoma.

Several investigations have evaluated the variations in gut microbiota composition in mouse models and hematologic patients (Figure 2). Moreover, the microbiota composition could change depending on the specific type of hematologic cancer (Supplementary Table S1; Riley et al., 2013; Allegra et al., 2019).

### Acute lymphoblastic leukemia

The role of the gut microbiota in acute lymphoblastic leukemia (ALL) the development remains unclear and is currently under investigation. Reports have identified variations in the GM composition profile in ALL patients compared to a healthy population. Other studies have shown a reduction in the relative abundance of *Edwardsiella tarda* and *Prevotella maculosa* in ALL patients, which was positively correlated with interleukin-10 levels (Kostic et al., 2013; Schirmer et al., 2016; Li et al., 2019; Liu et al., 2020).

Another study reported that *Faecalibacterium* abundance was reduced among ALL patients and negatively correlated with interleukin-6 (IL-6) and C-reactive protein (CRP) (Chua et al., 2017). Similarly, *Megamonas* was abundant in the gut microbiota of ALL children and correlated with the systemic inflammatory cytokines IL-6 (Sakon et al., 2008; Cozen et al., 2013; Bai et al., 2017; Li et al., 2018; Neisi et al., 2019; Ansari et al., 2021).

Furthermore, NGS analyses have revealed changes in microbiota diversity in ALL individuals, with an increase in Bacteroidetes and a decrease in Firmicutes. These alterations may be detrimental to leukemia patients. The Firmicutes phylum is the principal producer of butyrate (Venegas et al., 2019), which has been shown to have anti-cancer activities (Geng et al., 2021). For instance, researchers reported a significant reduction in butyrate production by the GM. Additionally, they found intestinal barrier damage in leukemia patients, which accelerated lipopolysaccharide (LPS) leakage into the bloodstream (Wang et al., 2022). LPS has been associated with leukemia progression both *in vivo* and *in vitro*. Butyrate is produced by certain bacteria such as *Eubacterium*, *Streptococcus*, *Clostridium*, *Bacteroides*, *Roseburia*, *Coprococcus*, *Ruminocococcus*, and *Butyrivibrio* (Ramsay et al., 2006; Anshory et al., 2023; Singh et al., 2023). Butyrate can repair the damage in the intestinal barrier, inhibiting LPS leakage and potentially playing a protective role against leukemia progression (Wang et al., 2022).

### Chronic lymphocytic leukemia

A common feature of chronic lymphocytic leukemia (CLL) is chronic systemic inflammation, with reports suggesting that dysbiosis may contribute to inflammation (Kawari et al., 2019). In the immune microenvironment of the intestine, T helper 17 cells (Th17) play an important role. Several studies demonstrate that increased levels of Th17 are an unfavorable prognostic factor in CLL. Huang et al. (2020) propose that *Prevotella* induces Th17 cell production in the mouse colon, highlighting its potential role in intestinal immune system formation (Huang et al., 2020).

Another study found that in patients with CLL, the most abundant bacteria were *Bacteroides*, *Parabacteroides*, *Prevotella*, and *Acinetobacter,* while there was *a* depletion of *Lachnospiraceae* and *Ruminococcaceae* (Faitová et al., 2022). In contrast, one study reported an increase in the abundance of Firmicutes and a decrease in Bacteroidetes compared to healthy individuals (Kawari et al., 2019).

The decrease in *Lachnospiraceae* and *Ruminococcaceae* may have several consequences for leukemia development (Vacca et al., 2020; Masetti et al., 2021). *Lachnospiraceae* has been associated with resistance to high radiation doses, hematopoiesis restoration, and butyrate-mediated repair of the gastrointestinal system in the host (Ma et al., 2021). Furthermore, studies have reported that the abundance of *Lachnospiraceae* is correlated with reduced side effects in patients with graft versus host disease (GVHD) (Ma et al., 2021).

*Ruminococcus* is another bacterium that produces several SCFAs (Mirzaei et al., 2021), and its deficit is associated with disruptions in several signaling pathways (Mirzaei et al., 2021). While the mechanisms of *Ruminococcaceae* in improving patient outcomes in leukemia are still unknown, there is evidence of increased *Ruminococcaceae* abundance in patients who achieved complete remission after PD-1 immunotherapy and CAR T-cell therapy (Ma et al., 2021; Zhou et al., 2022). Hence, *Ruminococcaceae* and its metabolites could improve the diagnosis and treatment of several cancer types.

### Acute myelogenous leukemia

Researchers have studied the role of gut microbiota in acute myelogenous leukemia (AML) by examining the differences in microbiota with and without treatment. One study published by Wang et al. (2022) reported a decrease in the gut microbiota diversity of AML patients. Moreover, the study found that intestinal damage was correlated with an increase in lipopolysaccharide levels and AML progression. Regarding bacterial species, the authors found that the reduction of *Faecalibacterium* could be involved in the proliferation and invasion of tumor cells and suppression of apoptosis (Ma et al., 2020; Wang et al., 2022).

Research suggests that most *Faecalibacterium* strains are associated with energy production for intestinal epithelial cells and the synthesis of metabolites, such as butyric acid, bioactive peptides, and anti-inflammatory substances, which contribute to intestinal health (Zou et al., 2021). Butyric acid modulates signaling pathways by interacting with the proinflammatory nuclear transcription factor NF-kB and inhibiting histone deacetylase (Knudsen et al., 2018). The regulation of metabolites, such as butyrate, could be an alternative for AML therapy development.

### Chronic myelogenous leukemia

According to research, chronic myelogenous leukemia (CML) patients have a higher abundance of Actinobacteria, Acidobacteria, and Chloroflexi, as well as a decreased abundance of Tenericutes. Furthermore, studies have described an increase in the levels of the *Streptococcus* genus in patients with CML compared to control patients (Yu et al., 2021). Several studies suggest an association between *Streptococcus* bacteria and an increase in the proinflammatory cytokine interferon γ (Bagheri et al., 2022). *Streptococcus* is essential in the sugar fermentation process, producing lactic acid as the main compound, which could have implications for CML progression (van den Bogert et al., 2013). Therefore, an imbalance of microbiota components could lead to proinflammatory responses, potentially triggering carcinogenesis (Liu et al., 2021).

An increased *Streptococcus* abundance may have a deleterious effect on leukemias, whereas the Actinobacteria abundance may help to decrease the adverse effects. Research has shown that the Actinobacteria phylum may benefit acute leukemia patients, as it is positively associated with Allo-HSCT immunotherapy (Ma et al., 2021) and exhibits antioxidant activities (Almuhayawi et al., 2021). Several Actinobacteria metabolites, such as indolocarbazoles, isoprenoids, non-ribosomal peptides, anthracyclines, macrolides, and enediynes, exhibit antioxidant and antitumoral properties. These metabolites have shown cytotoxic activity against cancer cell lines by reducing cyclooxygenase and lipoxygenase activity (Zhou et al., 2017; Almuhayawi et al., 2021). Cyclooxygenase is involved in prostaglandin synthesis, which promotes the proliferation of leukemia cells and the production of reactive oxygen species, while lipoxygenase catalyzes the production of hydroxyl eicosatetraenoic acids and leukotrienes, contributing to apoptosis suppression and the stimulation of tumor cell proliferation (Almuhayawi et al., 2021).

### Lymphomas

Understanding the correlation between gut microbiota, adaptive and innate immunity, and diseases like Hodgkin’s lymphoma is essential. Yuan et al. (2021) characterized the gut microbiota of 25 untreated individuals with diffuse large B cell lymphoma. Compared to the control group, the authors observed a higher abundance of Proteobacteria at the phylum level, as well as *Escherichia coli* (*E. coli*) and *Clostridium butyricum* (*C. butyricum*) species.

Various analyses have suggested that an increased prevalence of the bacterial phylum Proteobacteria could serve as a potential marker for an unstable microbial community (Shin et al., 2015; Tang et al., 2019) and be associated with B-cell differentiation (Yuan et al., 2021). Unlike most microbes, which are strict anaerobes, Proteobacteria are frequently facultatively or obligate anaerobic, enabling them to tolerate a wide range of toxic conditions.

On the other hand*, E. coli* produces colibactin and cytolethal-distending toxins, which have been associated with DNA breaks in epithelial cells, promoting genetic mutations and contributing to tumor formation. *E. coli* plays a crucial role in lymphoproliferative processes and infections by primarily colonizing the mucosal layer of the gastrointestinal tract, where it can contribute to chronic inflammation. Inflammation can persist due to these bacteria’ immune evasion strategies, including blocking TLR-4 signaling, NF-κB activity, and proinflammatory cytokines production in cells (Olson et al., 2014; Conway and Cohen, 2015; Rolhion and Chassaing, 2016).

Moreover, *C. butyricum*, a bacterium that produces butyrate and acetate, has been studied for its potential therapeutic use in dysbiosis-related diseases (Li et al., 2022). *C. butyricum* can also slow tumor growth by modulating Wnt/β-catenin signaling, which leads to decreased proliferation, and increased apoptosis (Tomita et al., 2022).

MALT lymphoma has been associated with a *Helicobacter pylori* infection, which could be involved in tumorigenesis and a chronic inflammatory response (Wotherspoon et al., 1991; O’Rourke, 2008; Saito et al., 2012; Moleiro et al., 2016). A retrospective study by Moleiro et al. (2016) showed that *H. pylori* eradication therapy could be effective for complete remission in patients (Moleiro et al., 2016).

### Multiple myeloma

Recent findings have shown an association between gut microbiota and MM (Lax et al., 2014; Alkharabsheh et al., 2020; Shapiro et al., 2021). Zhang et al. (2019) found that *Pseudomonas aeruginosa* and *Clostridium leptum* (*C. leptum*) were more abundant in MM patients. Moreover, higher levels of *C. leptum* were observed in MM patients with advanced stages of the disease. *Pseudomonas aeruginosa* can cause bacterial infections, while *C. leptum* is involved in the intestinal glucose metabolism pathway. Therefore, further research on these bacteria is critical for a better understanding of their roles (Zhang et al., 2019).

*Clostridium leptum* regulates glucose concentration in the intestinal microenvironment by producing butyrate through the pyruvate and acetyl-coenzyme A pathway. Butyrate plays a role in increasing regulatory T cells and suppressing interleukin 17 (IL-17) (Linares and Hermouet, 2022). For instance, Calcinotto et al. (2018) showed that a lack of IL-17 in MM mice, or treatment with antibiotics or antibodies that block IL-17/IL-17R interactions, leads toa delay in MM progression. The study identified *Prevotella heparinolytica* as the causal bacteria for IL-17 proliferation (Calcinotto et al., 2018). Therefore, the presence of butyrate-producing bacteria in the intestinal microbiota of MM patients is positively correlated with higher rates of minimal residual disease (MRD) negativity (Brevi et al., 2022).

Furthermore, Pianko et al. (2019) analyzed the microbiota composition of MRD in MM patients and found that MRD-negative treatment response was associated with a higher abundance of *Eubacterium hallii* and *F. prausnitzii*. *Eubacterium hallii* produces propionate, while *F. prausnitzii* produces butyrate. Both metabolites modulate immunity through autoinflammatory functions (Pianko et al., 2019).

## Discussion

The evidence presented in this mini-review underscores the role of specific microorganisms in the progression of hematologic diseases, given that microbiota imbalances have been found in all types of HC. Each type of HC —myeloma, lymphoma, and leukemia— exhibits distinct microbiota characteristics. Myeloma is characterized by an increased abundance of *Pseudomonas aeruginosa* and *Clostridium leptum*; lymphoma is associated with a higher proportion of *E. coli* and *C. butyricum*, while leukemia is marked by a decrease in *Lachnospiraceae* and *Ruminococcaceae*. These bacteria interact with immune cells in the epithelial tissue through their antigens or by secreting metabolites, potentially influencing the tumor environment. While these findings offer valuable insights, it is crucial to acknowledge that other factors and mechanisms may also contribute to cancer progression, warranting further investigation of the role and interactions of the gut microbiota with the tumor environment (Arthur et al., 2017). Notably, gut microbiota modulation may play a significant role in immune and treatment outcomes (Matson et al., 2018).

Microbiota modulation can be influenced by various factors, which may increase the risk of cancer development (De Agüero et al., 2016). Early interactions between the newborn, the mother, and the environment, such as the delivery and feeding methods, play a pivotal role in shaping the microbial microenvironment and long-term cancer susceptibility. Additionally, diet represents a critical factor that can be modified to prevent an imbalance of beneficial bacteria. Microbial food fermentation produces primary metabolites that can have either beneficial or detrimental effects on the host. Ongoing large-scale clinical trials are actively evaluating the efficacy of microbiota modulation, including dietary interventions and intratumoral injection of engineered bacteria (Sepich-Poore et al., 2021), as potential therapies for hematologic malignancies.

A comprehensive analysis of the microbiota concerning cancer may support disease management and deepen our understanding of host-microbial evolution. It also holds promise in exploring the microbiota as a distinguishable marker for cancer progression (Kalia et al., 2022). Fecal microbiome transplantation (FMT) is an alternative for restoring healthy microbiota in patients with hematologic diseases (Zheng et al., 2020). However, the characteristics of a healthy microbiome remain undefined, which leads to ongoing evaluation of FMT’s effectiveness in treating hematologic cancer, along with challenges like optimizing fecal processing and ensuring patient safety.

One of the main limitations of this research is that it relies on cross-sectional studies, limiting the capacity to establish a cause-effect relationship between microbiota and HC. Therefore, conducting longitudinal studies that measure the microbiota at different time points is essential for gain a comprehensive understanding of this interaction (Vogtmann and Goedert, 2016; Hou et al., 2022). There are other limitations, such as small sample sizes, ethnic bias, and the absence of control groups or disease staging in some studies. Moreover, technical limitations are also present as different techniques were used to identify microorganisms, resulting in the inability to capture the full complexity of the intestinal microbiota, potentially missing rare or less abundant species.

Furthermore, variations in the microbiome across different geographical regions should be considered. Characterizing microbiotas from diverse areas is essential to identify their primary composition. Moreover, it is crucial to carefully account for confounding factors such as diet, medication use, and the environment, as they could significantly impact the composition of the microbiota and its association with cancer progression (Fontana et al., 2019; Dwiyanto et al., 2021).

In conclusion, this mini review emphasizes the crucial role of specific microorganisms in hematologic cancer progression and highlights the significance of modulating the microbiota in immune responses and treatment outcomes. However, further research is essential to explore and comprehend the complexities of interactions between the gut microbiota and the tumor environment. Such studies are crucial for the development of targeted and effective microbiota-focused anticancer strategies, holding great promise for the future of hematologic cancer treatments.

## Author contributions

PG-R and AZ: conceptualization and writing – review and editing. SC-U: writing – original draft. EP-C, RT-T, and VR-P: investigation. AZ: supervision. All authors contributed to the article and approved the submitted version.

## Funding

The publication of this article will be funded by Universidad UTE-Ecuador. The funder had no role in the study design, bibliographic analysis, decision to publish, or preparation of the manuscript.

## Conflict of interest

The authors declare that the research was conducted in the absence of any commercial or financial relationships that could be construed as a potential conflict of interest.

## Publisher’s note

All claims expressed in this article are solely those of the authors and do not necessarily represent those of their affiliated organizations, or those of the publisher, the editors and the reviewers. Any product that may be evaluated in this article, or claim that may be made by its manufacturer, is not guaranteed or endorsed by the publisher.
