# Supplementary material for: Role of the gut microbiota in hematologic cancer
Source: Front Microbiol. 2023 Aug 25;14:1185787. doi: 10.3389/fmicb.2023.1185787 (PMC10485363; doi:10.3389/fmicb.2023.1185787)
Supplement: Supplementary file 1 [file Table_1.DOCX]

**Supplementary Tables**

**Supplementary Table 1.** Microbiota composition in hematologic cancer and the mechanism involved.

| **Hematologic Cancer** | **Microorganisms suspect to be involved** | **Mechanism** | **Metabolites** | **References** |
| --- | --- | --- | --- | --- |
| **Leukemias** | | | | |
| Acute lymphoblastic leukemia (ALL) | Phylum   - Decrease in Firmicutes - Increase in Bacteroidetes   Genus   - *Edwardsiella* and *Prevotella* - *Faecalibacterium* - *Megamonas* | - Lactic acid-producing bacteria - Fermentation of complex carbohydrates - Affecting Immune function (IL-10) - IL-6 and CRP - Cytokines IL-6 | - Butyrate is produced by Firmicutes and *Faecalibacterium* - Acetate and propionate are produced by Bacteroidetes and *Megamonas.* - Prevotella generates acetate - *Edwardsiella* generates trimethylamine N-oxide | (63–68,121–124) |
| Chronic lymphoblastic leukemia (CLL) | Phylum   - Decrease in Bacteroidetes and increase in Firmicutes   Phylun and family   - Abundant Bacteroidetes and depletion of *Lachnospiraceae* and *Ruminococcaceae* | - Immune response - Immune response | - Butyrate is produced by Firmicutes, *Lachnospiraceae* and *Ruminococcaceae* - Acetate and propionate are produced by Bacteroidetes | (50,75,77,124) |
| Acute myelogenous leukemia (AML) | Phylum  -Abundance of Actinobacteria*,* Acidobacteria, and Chloroflexi, lower abundance of Firmicutes and Tenericutes  Genus   - Decrease in *Faecalibacterium* | - Unspecified - Immune response | - Butyrate is produced by Firmicutes and *Faecalibacterium* - Actinobacteria produces indolocarbazoles, isoprenoids, non-ribosomal peptides, anthracyclines, macrolides, and enediynes | (82,83,86,90,91,124) |
| Chronic myelogenous leukemia(CML) | Phylum   - Abundance of Actinobacteria, Acidobacteria, and Chloroflexi and lower abundance of Tenericutes   Genus   - Increase in *Streptococcus* | - Unspecified - More studies of its relationship with cancer progression | - Actinobacteria produces indolocarbazoles, isoprenoids, non-ribosomal peptides, anthracyclines, macrolides, and enediynes - *Streptococcus* generates acetate | (86,88,90,124) |
| **Lymphomas** | | | | |
| Hodgkin´s Lymphoma | - Modest microbiota reduction in patients compared to controls | - More studies are needed |  | (125) |
| Non – Hodgkin´s Lymphoma | Phylum   - Abundance of Proteobacteria   Species   - Abundance of *E. coli* - Abundance of *C. butyricum* | - B cell differentiation   - Tumor formation   - Unspecified | - *C. butyricum* generate butyrate and acetate - *E. coli* generate isopropanol | (94,99,124) |
| **Myeloma** | | | | |
| Multiple Myeloma | Species   - Abundance of *Pseudomonas aeruginosa* - Abundance of *Clostridium leptum* | - Related to the pathogenic species - Involved in the intestinal glucose metabolism | - Butyrate is produced by *Clostridium leptum* | (107,108,124) |
